# Supplementary material for: Phosphates Induced H-Type or J-Type Aggregation of Cationic Porphyrins with Varied Side Chains
Source: Molecules. 2023 May 16;28(10):4115. doi: 10.3390/molecules28104115 (PMC10223173; doi:10.3390/molecules28104115)
Supplement: Supplementary file 1 [file molecules-28-04115-s001.zip › molecules-2394808-supplementary.pdf]

Supporting Information

Phosphates Induced H-type or J-type Aggregation of Cationic Porphyrins with Varied Side Chains

Zhiliang Li [1,2] Charles J. Zeman IV [1,2] Silvano Valandro [1], Jose Paolo O. Bantang[1],

and Kirk S. Schanze [1,*]

1 Department of Chemistry, University of Texas at San Antonio, San Antonio, TX 78249, USA; zhiliang.li@sdu.edu.cn (Z.L.); charles.zeman@northwestern.edu (C.J.Z.IV); silvano.valandro@chemie.uni-hamburg.de (S.V.); jopaobantang@gmail.com (J.P.O.B.)
2 Department of Chemistry, University of Florida, Gainesville, FL 32611, USA

*Corresponding author: kirk.schanze@utsa.edu

1. Synthesis and Characterization

Synthesis of the phosphate-responsive porphyrin derivatives: Two chiral symmetric porphyrin derivatives featuring linear or branched ammonium side groups were synthesized according to the procedures described below.

General procedure 1 for the acid-amine coupling reaction:

A solution of the porphyrin-based tetrakis(acid) (1.0 eq.), N-(3-dimethylaminopropyl)-N′-ethylcarbodiimide hydrochloride (EDCI) (8.0 eq.), hydroxybenzotriazole (HOBt) (8.0 eq.), N,N-diisopropylethylamine (DIPEA) (24.0 eq.) in chloroform (10 mL) was stirred at room temperature (r.t.) for 1 h, followed by adding the respective amine (5.0 eq.). The resulting solution was stirred at r.t. in the dark for another 24 h in an N_2 atmosphere. The reaction mixture was diluted with dichloromethane (DCM) (10 mL) and then washed successively with satd. NH_4Cl (aq) (20 mL×2), satd. Na_2CO_3 (aq) (20 mL×2) and brine (20 mL×2). The organic phase was evaporated to dryness under reduced pressure after being dried over anhydrous Na_2SO_4. The residue was subjected to column chromatography (SiO_2, CH_2Cl_2/MeOH 30/1 v/v) to allow isolation of products as dark-red solids.

General procedure 2 for the deprotection reaction:

To a dioxane (10 mL) solution of the red solid obtained from procedure 1 was added a concentrated HCl solution with vigorous stirring at 0 ^{o}C. The mixture was allowed to warm slowly to room temperature and stirred in the dark for 24 h in an N_2 atmosphere. The reaction mixture was dropped into cold acetone (100 mL), and the green precipitate was collected by centrifugation. The green solid was redissolved in pH=2 deionized water (DIW) and precipitated in cold acetone (100 mL) again to obtain the final product as a green solid. The green solid was dissolved in DIW and adjusted to a concentration of 1 mM as stock solution.

Synthesis of porphyrin 1 (Scheme S-1):

Synthesis of compound 1-2. Compound **1-2** was synthesized according to the general procedure 1 using **1-1** (80 mg, 0.1 mmol), EDCI (150 mg, 0.8 mmol, 8.0 eq.), HOBt (135 mg, 0.8 mmol, 8.0 eq.), and DIPEA (0.45 mL, 2.4 mmol, 24.0 eq.) in chloroform (10 mL) to obtain the product as a dark red solid (80 mg, 0.071 mmol, 71%). ^{1}H NMR (500 MHz, $CDCl_3$, 25 °C) δ (ppm) 8.82 (s, 8H), 8.31 (d, J = 15.0 Hz, 8H), 8.22 (d, J = 15.0 Hz, 8H), 7.07 (d, J = 15.0 Hz, 4H), 5.00 (m, 4H), 3.89 (s, 12 H), 1.69 (d, J = 10.0 Hz, 12 H); ^{13}C NMR (125 MHz, $CDCl_3$, 25 °C) δ (ppm) 174.0, 167.1, 145.7, 134.8, 133.7, 125.8, 119.5, 53.0, 49.0, 19.0. HR-MS (ESI) mass: m/z; calculated for compound **1-2**, $C_{64}H_{58}N_8O_{12}$, [M + H^+], 1131.4247; Found: [M + H^+], 1131.4270.

Synthesis of porphyrin 1. Ester **1-2** (80 mg, 0.071 mmol) was dissolved in a mixed solvent of tetrahydrofuran (THF) (3 mL) and DIW (3 mL), followed by adding LiOH (24 mg, 1 mmol) in one portion. The resulting mixture was stirred at r.t. until TLC showed that the ester was completely consumed. The reaction was acidified with 1M HCl to pH=2. The green precipitate was collected by centrifugation and dissolved in pH=2 DIW and collected by centrifugation again. It was then dried in a vacuum oven to give a green solid (quantitative), which was subjected to reaction with amine **1-4** [1] for synthesis of compounds **1-3** and **1** according to the general procedure **1** and **2** described above for coupling reaction and deprotection. Finally, compound **1**, obtained as a green solid (60 mg, 0.046 mmol, 65% in 3 steps) was dissolved in DIW and adjusted to a concentration of 1 mM as stock solution.

Compound 1-3: ^{1}H NMR (500 MHz, CD_3OD, 25 °C) δ (ppm) 8.47 (br s, 8H), 8.04 (br s, 8H), 7.84 (br s, 8H), 4.70 (q, J = 15.0 Hz, 4H), 3.33 (m, 8H), 3.16 (t, J = 10.0 Hz, 8H), 1.72 (m, 8H), 1.52 (d, J = 10.0 Hz, 12 H), 1.41 (s, 36H); ^{13}C NMR (125 MHz, CD_3OD, 25 °C) δ (ppm)

174.8, 166.8, 143.9, 134.2, 134.0, 126.0, 119.5, 45.2, 17.8. HR-MS (ESI) mass: m/z; calculated for compound **1-3**, $C_{92}H_{114}N_{16}O_{16}$, [M + $2H^+$], 850.4372; Found: [M + $2H^+$], 850.4377.

Compound 1. ^{1}H NMR (500 MHz, CD_3OD, 25 °C) δ (ppm) 8.82 (s, 8H), 8.76 (d, J = 10.0 Hz, 8H), 8.60 (d, J = 10.0 Hz, 8H), 4.71 (q, J = 10.0 Hz, 4H), 3.49 (m, 8H), 3.13 (t, J = 10.0 Hz, 8H), 2.01 (m, 8H), 1.70 (d, J = 10.0 Hz, 12 H); ^{13}C NMR (125 MHz, CD_3OD, 25 °C) δ (ppm) 175.1, 168.3, 142.1, 138.2, 135.2, 129.1, 127.5, 122.0, 100.0, 50.7, 36.9, 35.6, 27.4, 16.6. HR-MS (ESI) mass: m/z; calculated for compound **1**, $C_{72}H_{82}N_{16}O_8$, [M + $3H^+$], 433.8907; Found: [M + $3H^+$], 433.8901.

Synthesis of porphyrin 2 (Scheme S-2):

Synthesis of compound 2-2: To a solution of Fmoc-Ala-OH (311 mg, 1 mmol) in anhydrous $CHCl_3$ (5 mL) was added 2 drops of dimethylformamide (DMF) and oxalyl chloride (381 mg, 3 mmol) successively at r.t. The resulting mixture was stirred under N_2 for 1 h, at which time the solution was clear yellow. The solvent and the excess oxalyl chloride were removed by dry N_2 flow to give a yellow solid, which was dissolved in anhydrous $CHCl_3$ (5 mL). Then, to the above carbonyl chloride solution was added to a solution of amine **4** [2] (500 mg, 1.2 mmol) in anhydrous $CHCl_3$ (5 mL) and DIPEA (0.5 mL) at 0°C with vigorous stirring. The reaction was slowly warmed to r.t. and stirred at r.t. for 24 h. Then the reaction was quenched with sat. aq. NH_4Cl and washed with sat. aq. Na_2CO_3 (50 mL) twice and brine (50 mL). The organic phase was dried over anhydrous $MgSO_4$ and the solvent was removed under reduced pressure after filtration. The crude product was purified by column chromatography (SiO_2, CH_2Cl_2/Ethyl Acetate 3/1 v/v) to allow isolation of product **2-2** as a yellow oil (200 mg, 0.532 mmol, 53.2%). ^{1}H NMR (500 MHz, $CDCl_3$, 25 °C) δ (ppm) 7.75 (d, J = 5 Hz, 2H), 7.60 (d, J = 5 Hz, 2H), 7.38 (t, J = 5 Hz, 2H), 7.29 (t, J = 5 Hz, 2H), 7.05 (br s, 1H), 5.77 (br s, 1H), 4.88 (br s, 3H), 4.37 (m, 2H), 4.20 (t, J = 5

Hz, 2H), 3.09 (m, 6H), 1.92 (m, 6H), 1.44 (d, J = 10 Hz, 3H), 1.40 (s, 27H); ^{13}C NMR (125 MHz, $CDCl_3$, 25 °C) δ (ppm) 172.6, 156.4, 144.1, 144.0, 141.5, 127.9, 127.3, 125.3, 120.2, 79.7, 67.2, 56.7, 51.5, 47.3, 36.4, 36.1, 29.9, 28.6. HR-MS (ESI) mass: m/z; calculated for compound **2-2**, $C_{40}H_{59}N_5O_9$, [M + H^+], 754.4386; Found: [M + H^+], 754.4383.

Synthesis of porphyrin 2: A 0.5 mL aliquot of piperidine was added to a solution of compound **2-2** (200 mg, 0.532 mmol) in anhydrous $CHCl_3$ (5 mL) at r.t. The reaction was stirred at r.t. for about 5 h, at which time TLC showed that the starting material was consumed completely. The solvent was removed under reduced pressure and the crude product was used without further purification for the coupling reaction with acid **1-1** as shown in scheme S-2, according to the general procedure **1** and **2** described above. Finally, compound **2**, obtained as a green solid (30 mg, 0.021 mmol, 34% in 3 steps), was dissolved in DIW and adjusted to a concentration of 1 mM as stock solution.

Compound 2: ^{1}H NMR (500 MHz, C D_3OD, 25 °C) δ (ppm) 8.91 (br, 8H), 8.54 (br, 8H), 8.47 (br, 8H), 4.61 (m, 4H), 3.18 (m, 24H), 2.29 (m, 24H), 1.68 (d, J = 10 Hz, 12H); ^{13}C NMR (125 MHz, CD_3OD, 25 °C) δ (ppm) 175.0, 169.6, 143.7, 136.6, 133.9, 130.5, 127.7, 126.9, 121.0, 55.4, 51.31, 34.6, 32.0, 16.8. HR-MS (ESI) mass: m/z; calculated for compound **2**, $C_{88}H_{122}N_{24}O_8$, [M + $4H^+$], 411.7542; Found: [M + $4H^+$], 411.7542.

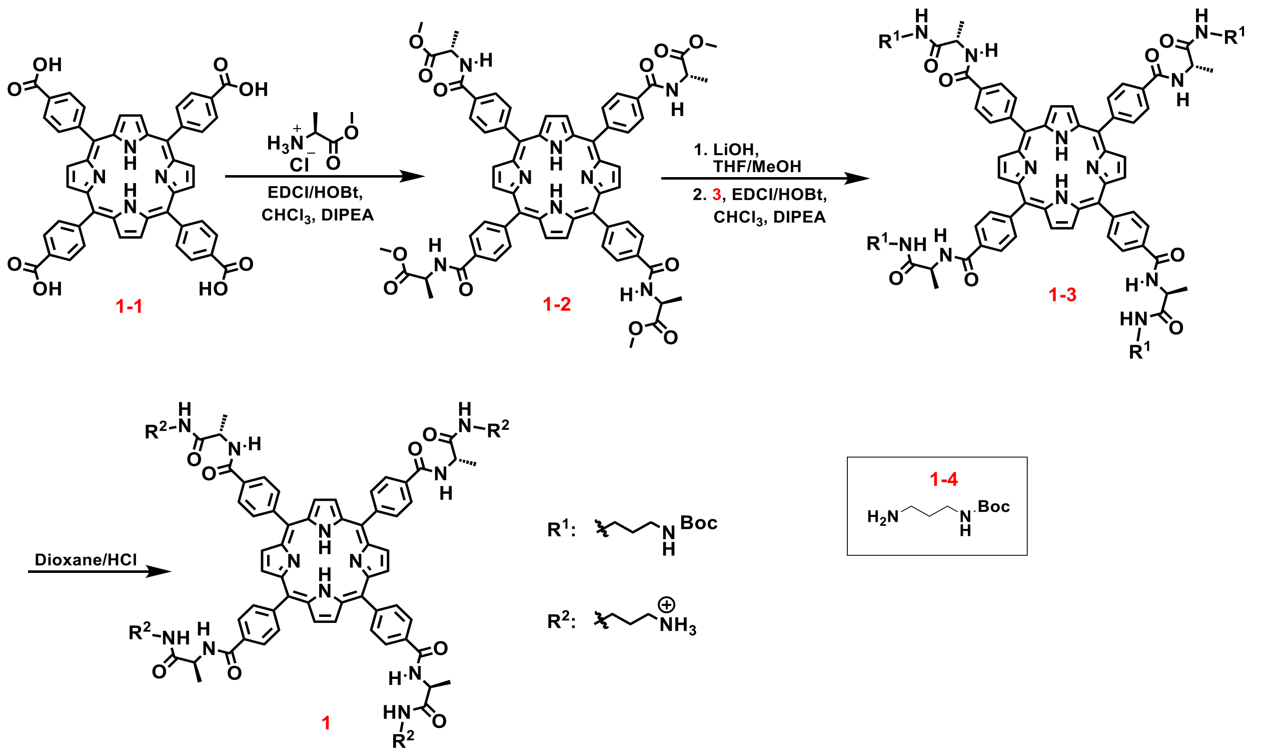

Figure S1. Synthesis scheme of porphyrin **1**.

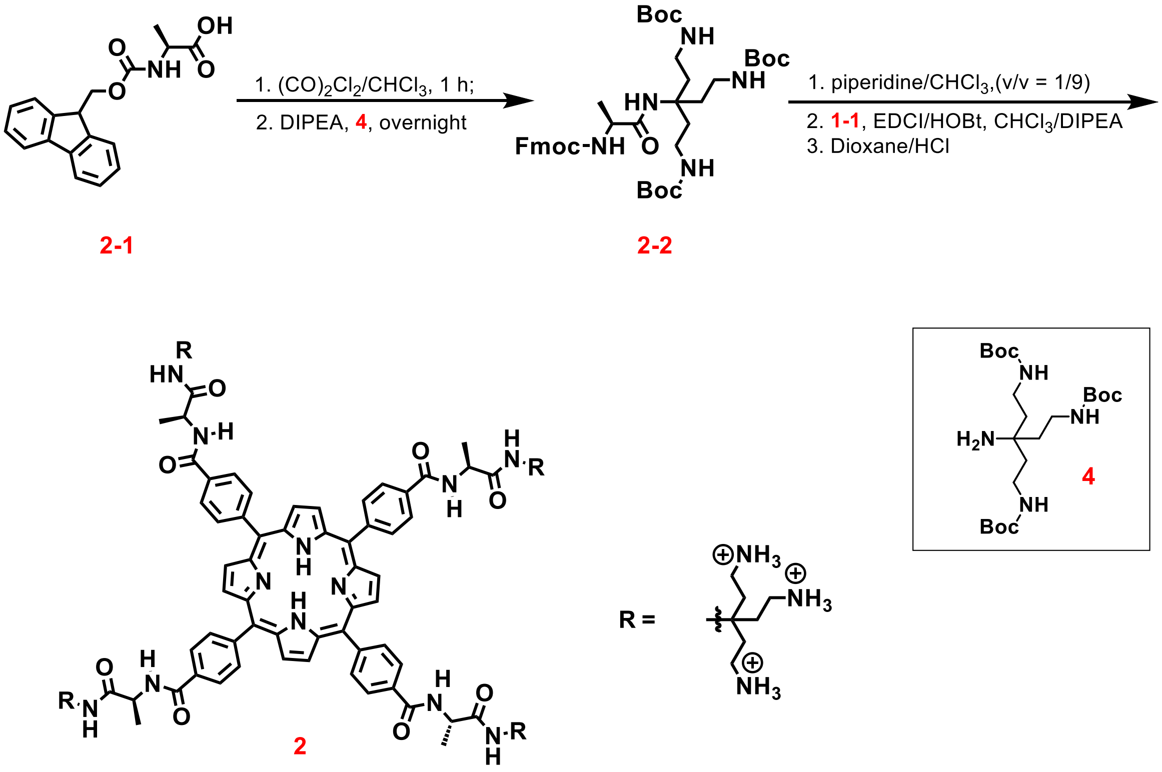

Figure S-2. Synthesis scheme of porphyrin **2**.

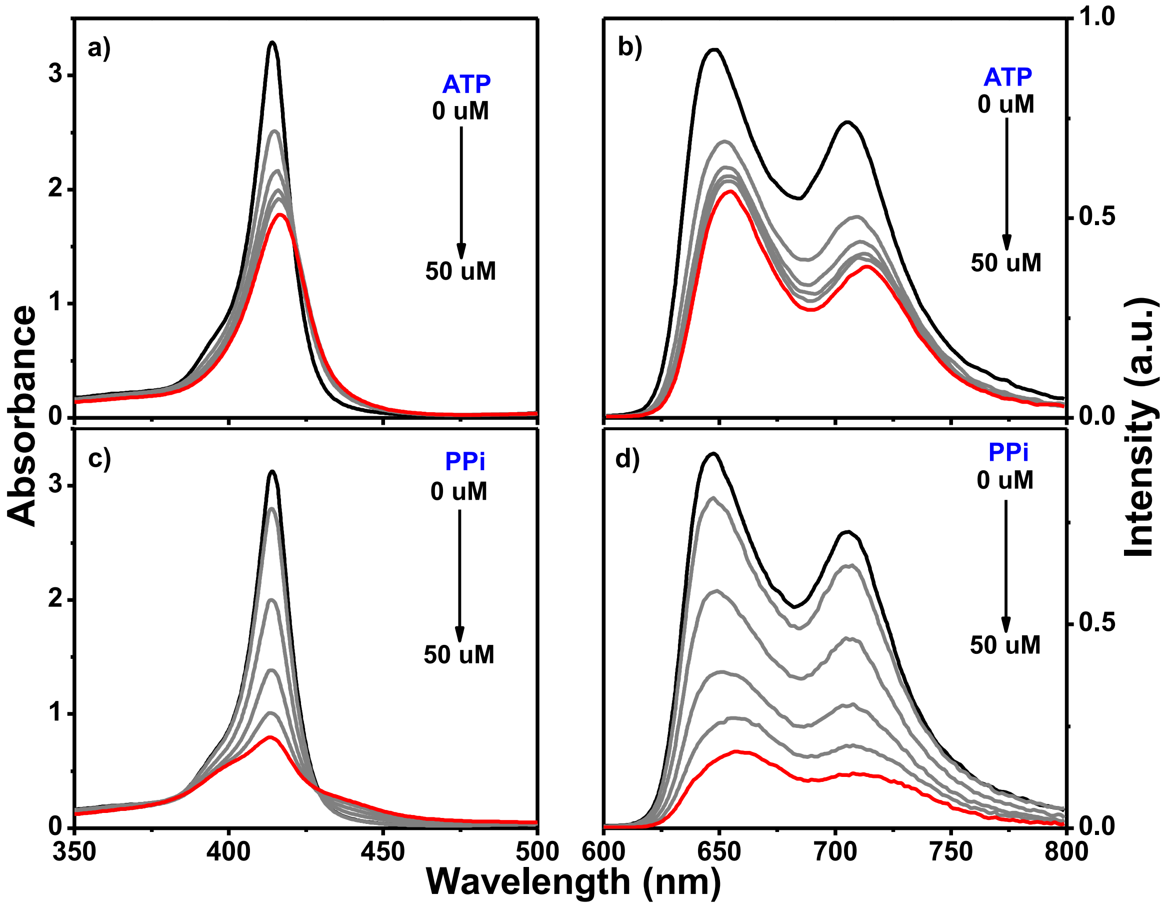

Figure S-3. Absorption spectra of **1** in DIW with increasing concentrations of ATP (a) and PPi (c). Fluorescence spectra of **1** in DIW with increasing concentrations of ATP (b) and PPi (d). ([**1**]: 10 μM in all cases).

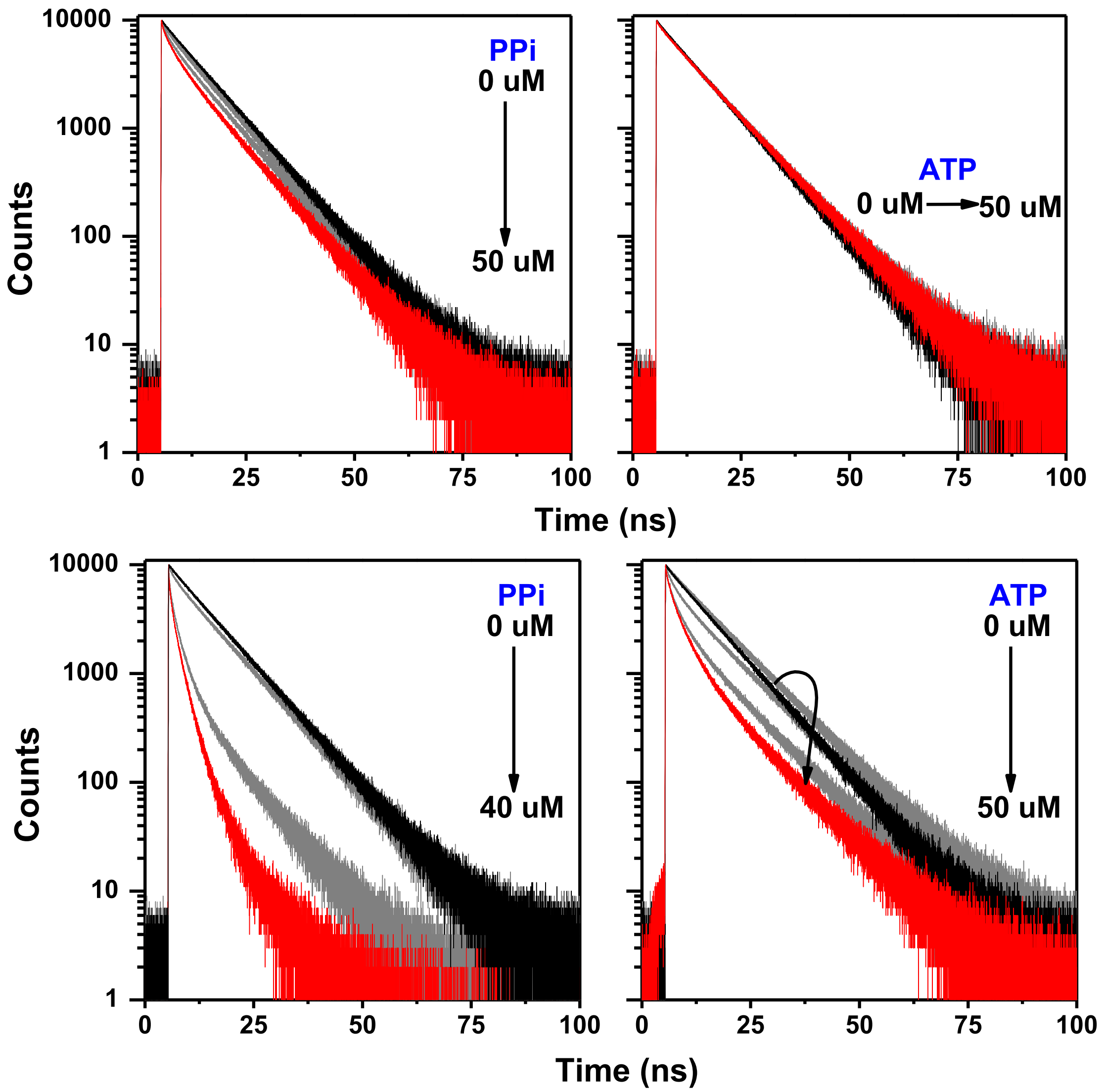

Figure S-4. Time-dependent fluorescence decay data of **1** (top) **and 2** (bottom) with increasing concentrations of PPi and ATP. **1** (10 μM in all cases) was excited at 515 nm and the emission was collected at 650 nm. **2** (10 μM in all cases) was excited at 515 nm and the emission was collected at 650 nm.

Table S-1. Fluorescence Lifetimes of Porphyrin **1** and **2** in free and aggregated states with phosphates.

Sample	Quencher	τ_1 (ns)	a_1 (Cnts)	τ_2 (ns)	a_2(Cnts)	τ_3 (ns)	a_3(Cnts)	χ^2
**1**	0uM	9.42±0.04	10708 (100%)	-	-	-	-	0.81
	10uM PPi	9.39±0.04	10407 (100%)	-	-	-	-	0.82
	20uM PPi	9.31±0.04	10400 (100%)	-	-	-	-	0.84
	30uM PPi	9.38±0.04	9470 (87%)	2.64±0.32	1386 (13%)	-	-	0.80
	40uM PPi	9.32±0.04	9172 (72%)	2.24±0.13	3593 (28%)	-	-	0.80
	50uM PP	9.20±0.04	7657 (58%)	2.59±0.09	5501 (42%)	-	-	0.81
	10uM ATP	9.60±0.04	10718 (100%)	-	-	-	-	0.89
	20uM ATP	9.95±0.04	9492 (91%)	4.00±0.52	984(9%)	-	-	0.82
	30uM ATP	10.04±0.04	9599(90%)	3.95±0.50	1016 (10%)	-	-	0.82
	40uM ATP	10.09±0.04	9569 (90%)	4.01±0.51	1007 (10%)	-	-	0.83
	50uM ATP	10.13±0.04	9425 (90%)	3.87±0.51	991 (10%)	-	-	0.81
**2**	0 uM	9.53±0.03	10592 (100%)	-	-	-	-	0.87
	10uM PPi	9.53±0.04	10657 (100%)	-	-	-	-	0.84
	20uM PPi	9.59±0.04	9501 (82%)	1.54±0.19	2032 (18%)	-	-	0.83
	30uM PPi	8.64±0.09	894 (6%)	2.17±0.03	4685 (30%)	0.28±0.01	9809 (64%)	0.73
	40uM PPi	5.23±0.08	536 (3%)	1.95±0.02	4811 (31%)	0.26±0.01	10247 (66%)	0.81
	50uM PPi	4.01±0.05	842 (4%)	1.71±0.02	5004 (22%)	0.21±0.01	16674 (74%)	0.82
	10uM ATP	10.48±0.04	10495 (100%)	-	-	-	-	0.84
	20uM ATP	10.62±0.05	10794 (100)	-	-	-	-	0.98
	30uM ATP	10.78±0.05	7358 (66%)	2.41±0.12	3850 (34%)	-	-	0.78
	40uM ATP	10.51±0.07	4170 (38%)	2.52±0.06	6909 (62%)	-	-	0.81

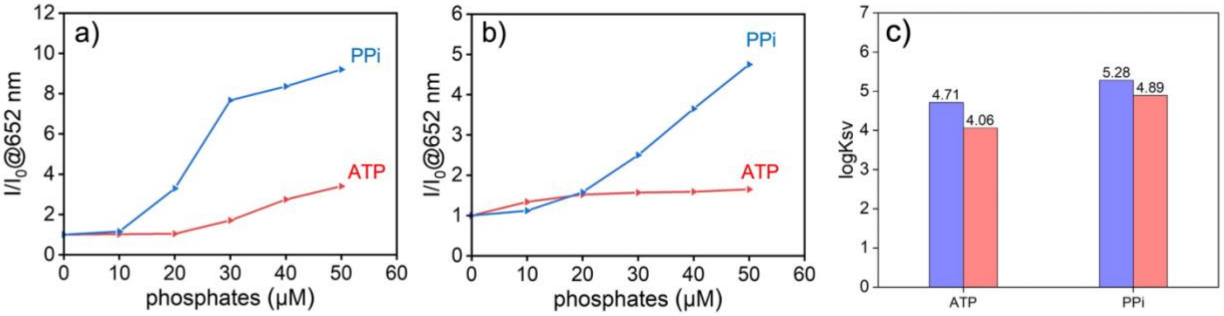

Figure S-5. Stern–Volmer plots of **1** (a) and **2** (b) with PPi and ATP as quenchers in 10 μM porphyrin solution in water. (c) Stern–Volmer constants for quenchers in fluorescence quenching of 1 (red) and 2 (blue) in 10 μM porphyrin solution in water. The intensities are monitored at the fluorescence band maximum for each porphyrin.

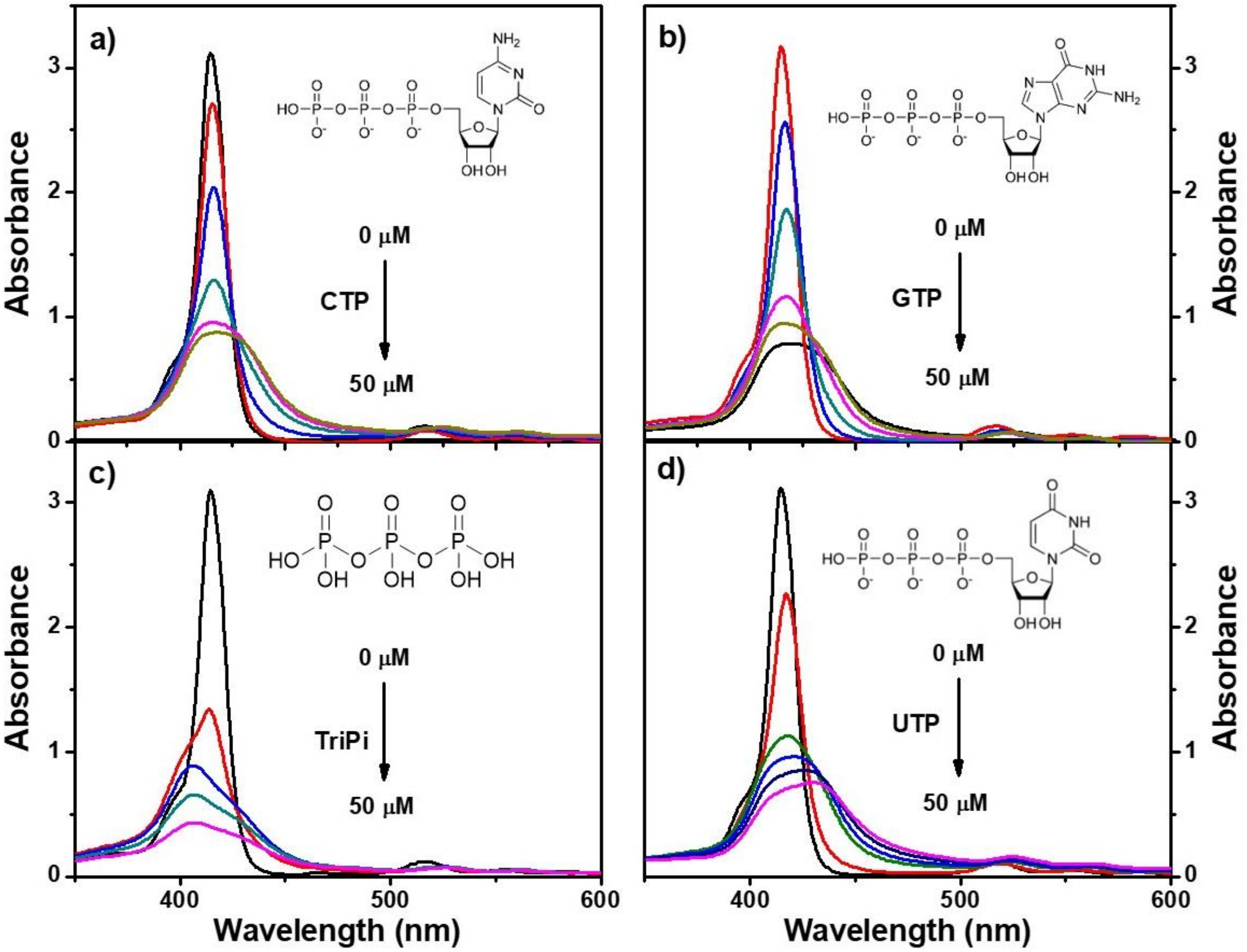

Figure S-6. Absorption spectra of **2** titration experiments with TriPi and nucleoside triphosphates (NTPs). ([**2**]: 10 μM)

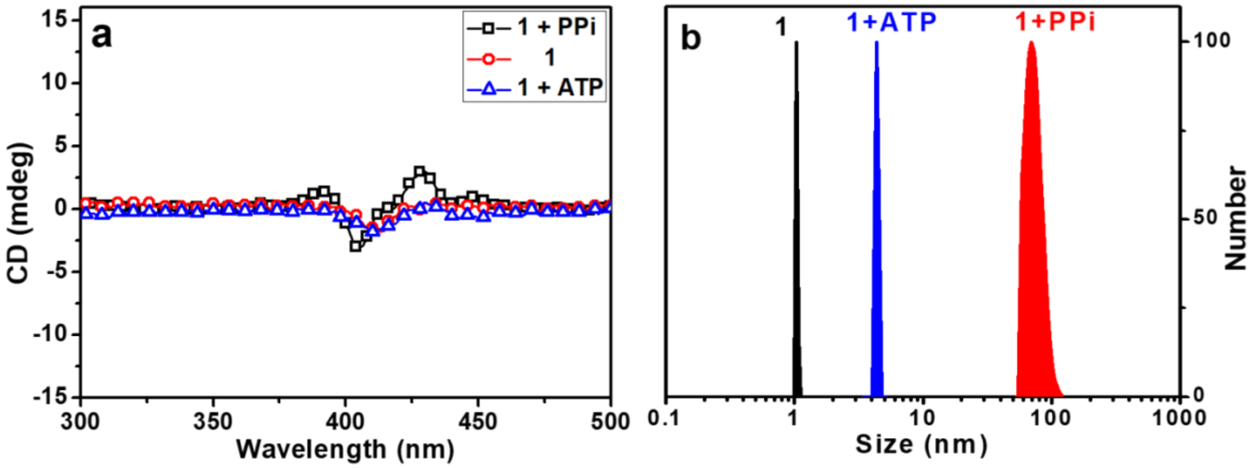

Figure S-7. Circular dichroism spectra (a) and DLS size distribution (b) of **1** and its assemblies with PPi or ATP. ([**1**]: 10 μM, [PPi] or [ATP]: 30 μM)

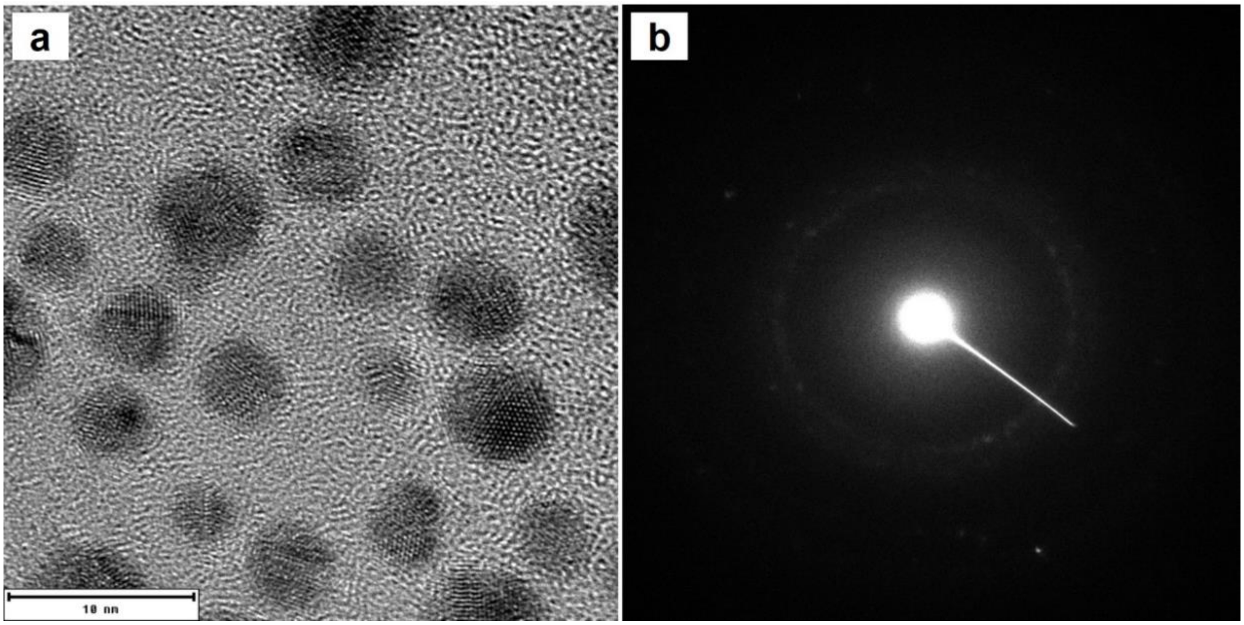

Figure S-8. HRTEM image (a) and SAED image (b) of **1** with 3.0 equivalent ATP. Scale bar: 10 nm. ([**1**]: 10 μM).

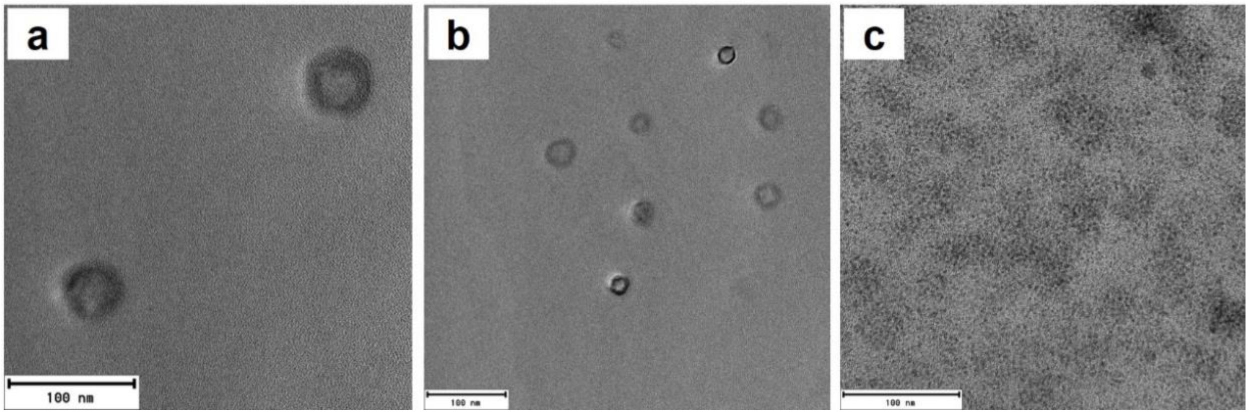

Figure S-9. HRTEM images of **1** with 3.0 equivalent PPi (a and b) or ATP (c). Scale bar: 100 nm. ([**1**] = 10 μM).

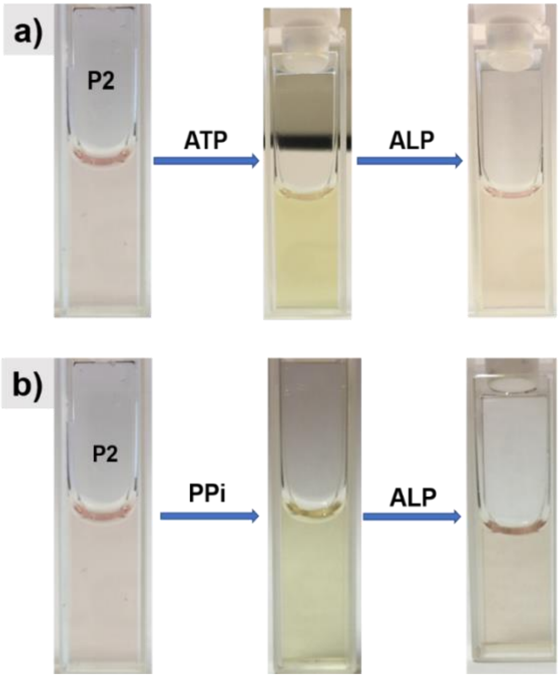

Figure S-10. Images that show color change of **2** solutions after adding ATP (a) and PPi (b) and the following color recovery after hydrolysis completed by ALP.

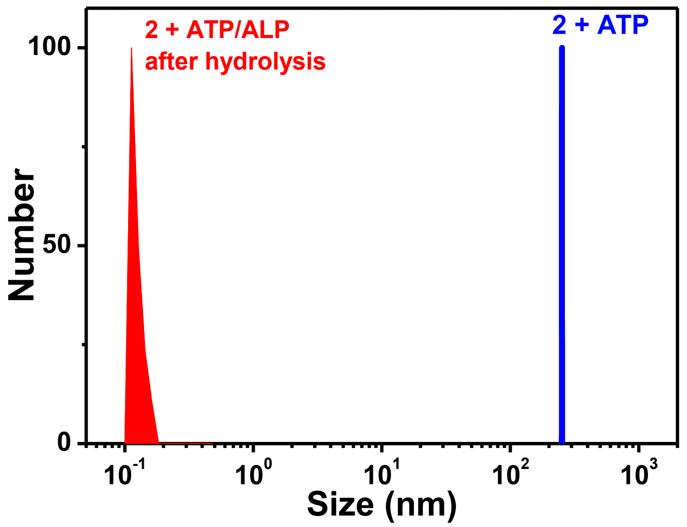

Figure S-11. DLS size distribution showing **2**-ATP assembly disassociation by ALP in MES buffer at 38 °C. ([**2**]: 10 μM, [ATP]: 30 μM, [ALP]: 1.5 U/mL, [MES]: 10 mM).

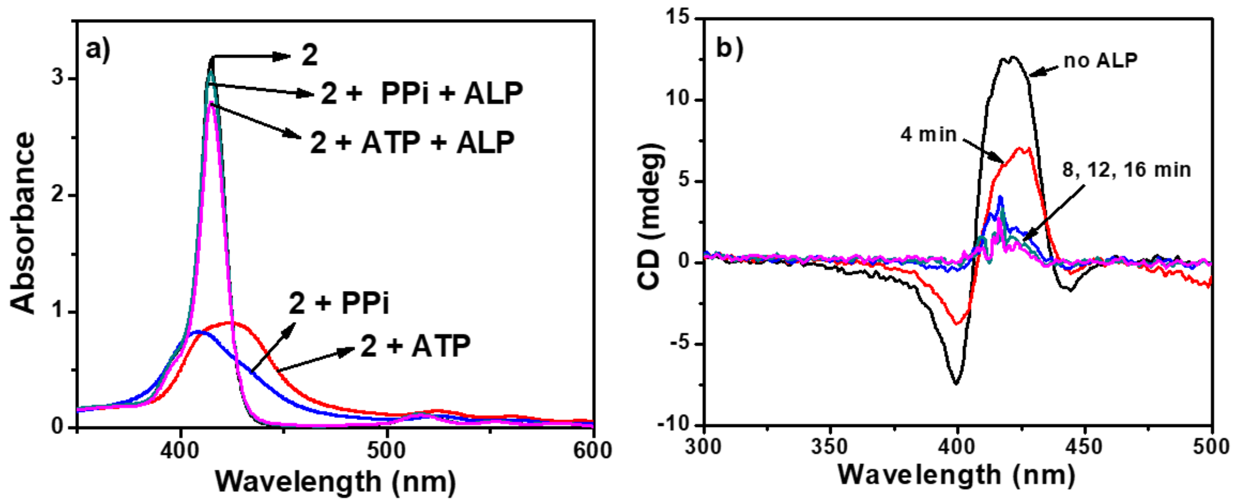

Figure S-12. a). Absorption spectra of **2** before and after addition of 30 μM PPi or ATP and after completion of hydrolysis catalyzed by ALP; b). Time-dependent CD spectra showing conformational reversal of **2**-PPi assembly with ALP at 38 °C. ([**2**]: 10 μM in all cases, [PPi]: 30 μM, [ATP]: 30 μM, [ALP]: 1.5 U/mL, [MES]: 50 mM)

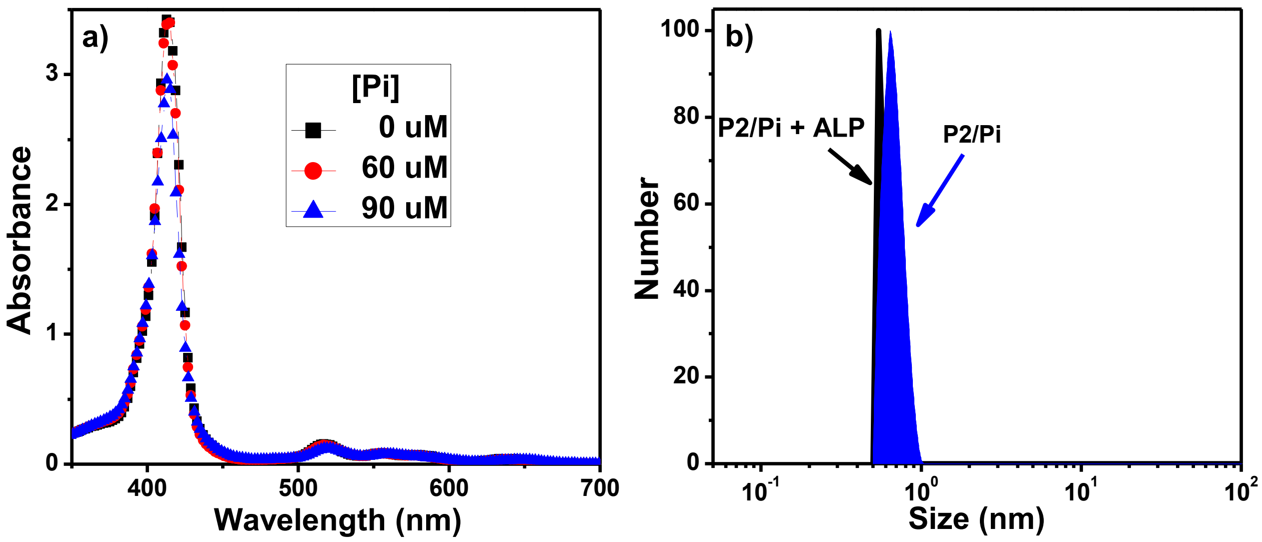

Figure S-13. a) Absorption spectra of 2 titration experiments with Pi; b) Number percentage DLS size changes before and after addition of Pi (60 μM) and ALP (1.5 U/mL) at 38 °C.

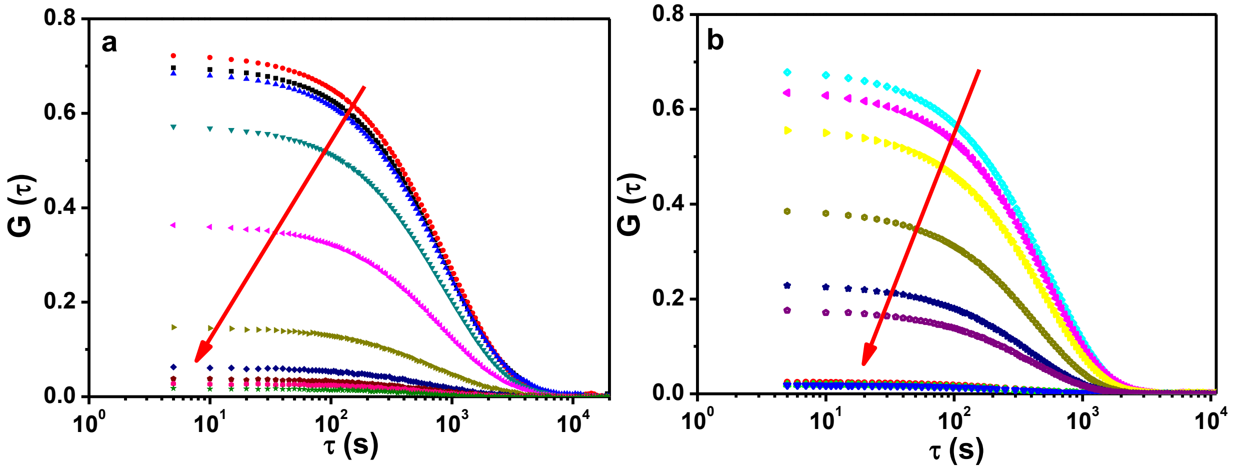

Figure S-14. Time-dependent DLS correlation function curves of **2**-PPi complex (a) and **2**-ATP complex (b) during hydrolysis by ALP (1.5 U/mL) in aqueous buffer solution recorded every 90 seconds. ([**2**]: 10 μM, [PPi]: 30 μM, [ATP]: 30 μM, [MES]: 50 mM, 38 °C).

2. NMR Spectra of New Compounds (^{1}H NMR, ^{13}C NMR)

1-2

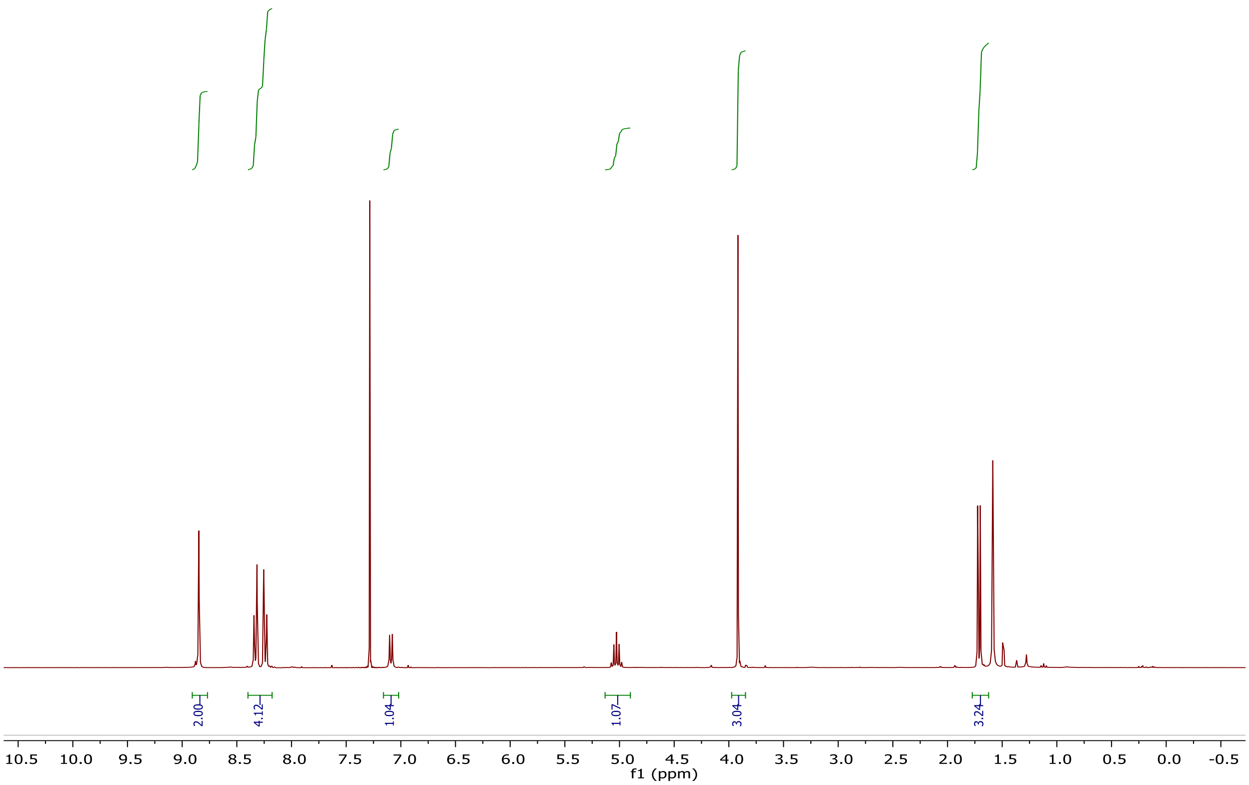

1-2

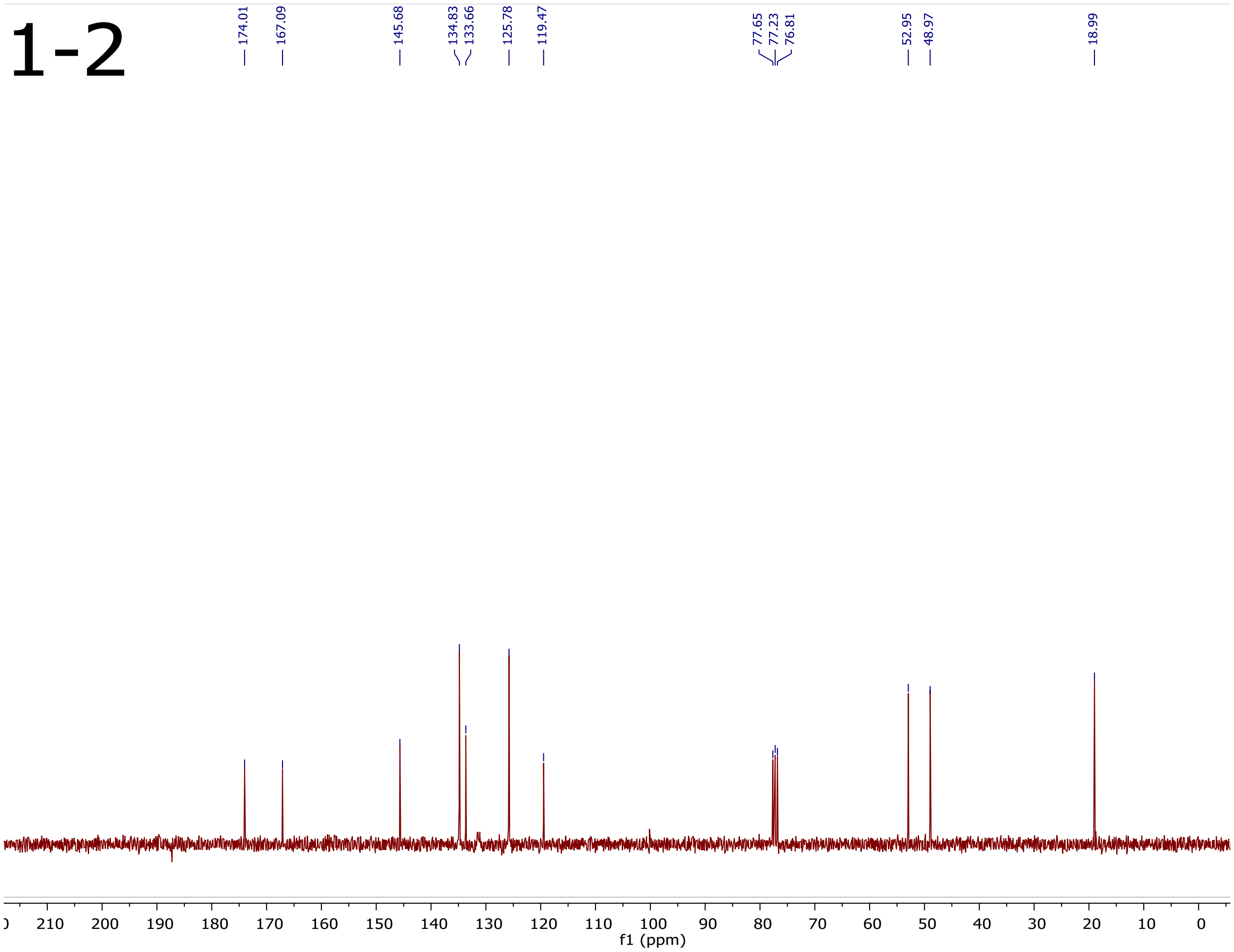

174.01
167.09
145.68
134.83
133.66
125.78
119.47
77.65
77.23
76.81
52.95
48.97
18.99
210 200 190 180 170 160 150 140 130 120 110 100 90 80 70 60 50 40 30 20 10 0
f1 (ppm)

1-3

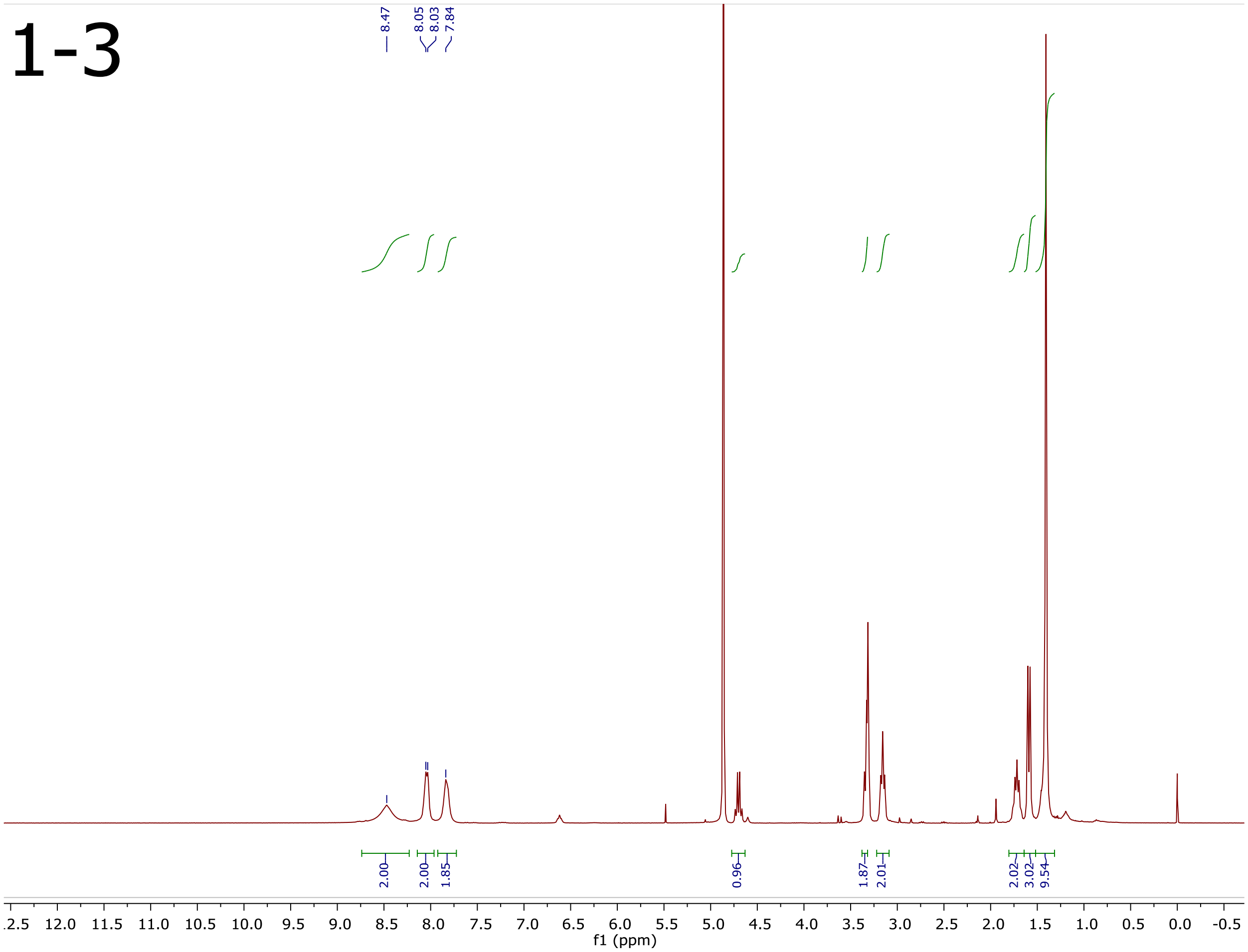

1-3

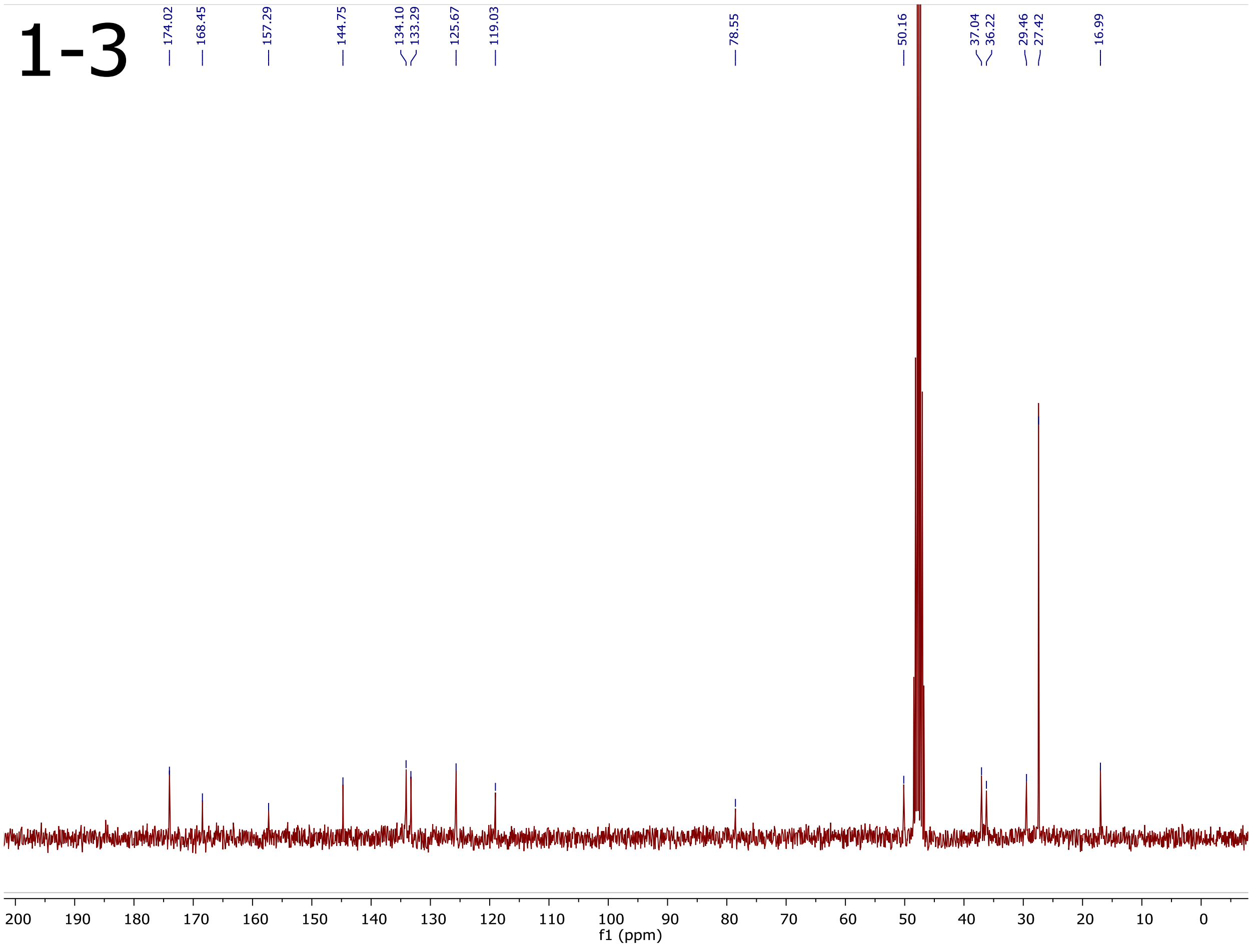
174.02
168.45
157.29
144.75
134.10
133.29
125.67
119.03
78.55
50.16
37.04
36.22
29.46
27.42
16.99
f1 (ppm)

1

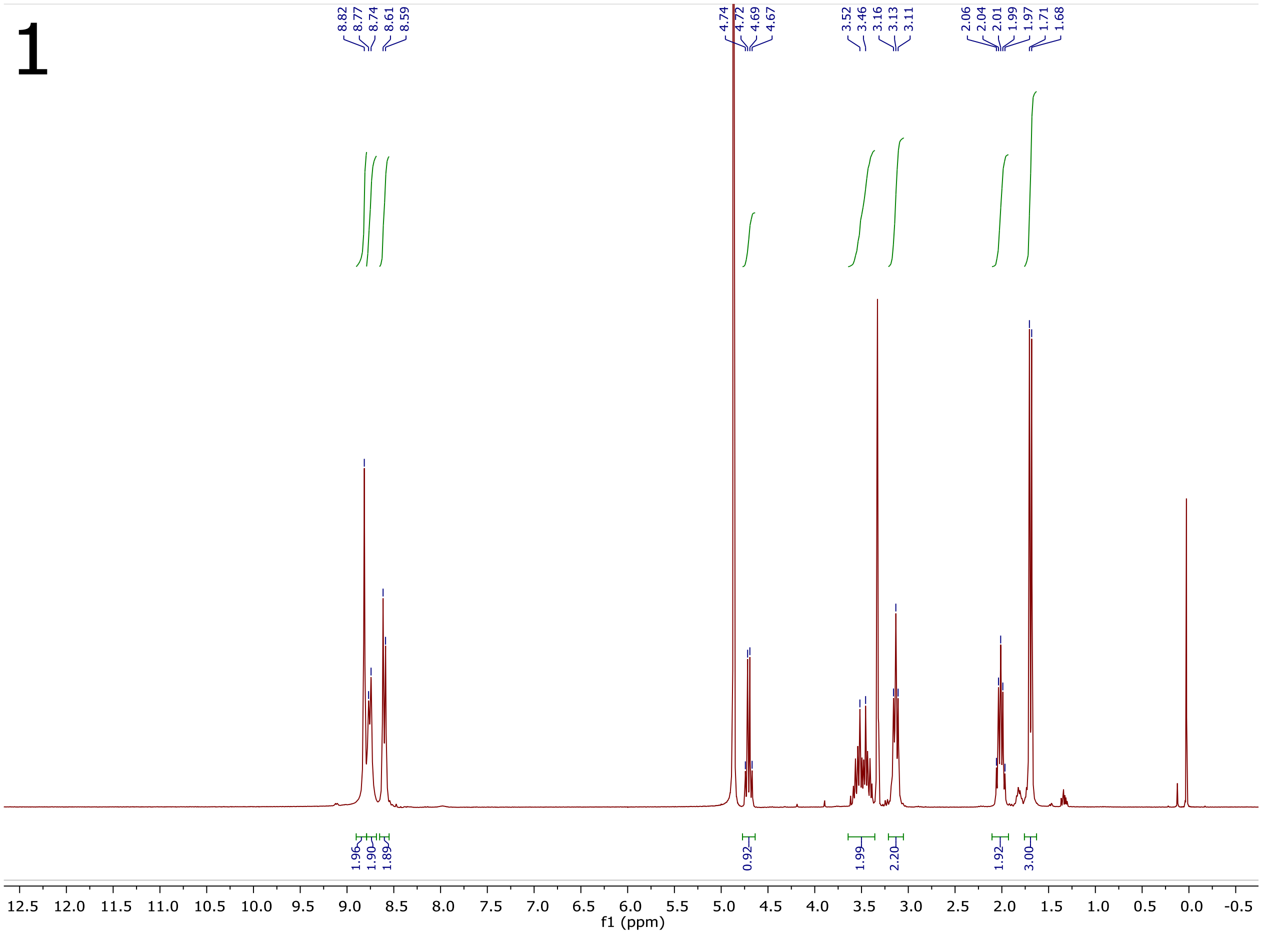

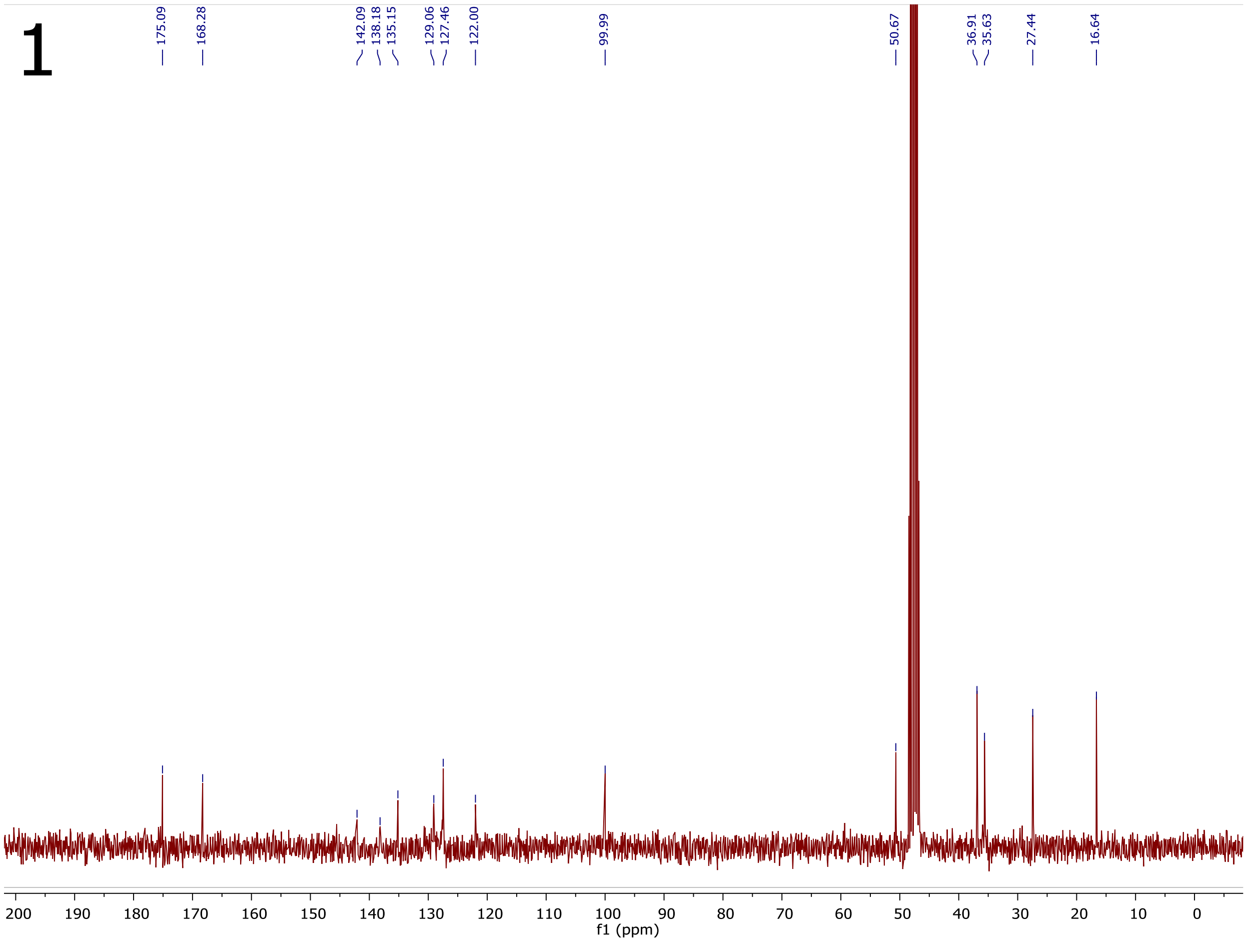
1
175.09
168.28
142.09
138.18
135.15
129.06
127.46
122.00
99.99
50.67
36.91
35.63
27.44
16.64
200
190
180
170
160
150
140
130
120
110
100
90
80
70
60
50
40
30
20
10
0
f1 (ppm)

2-2

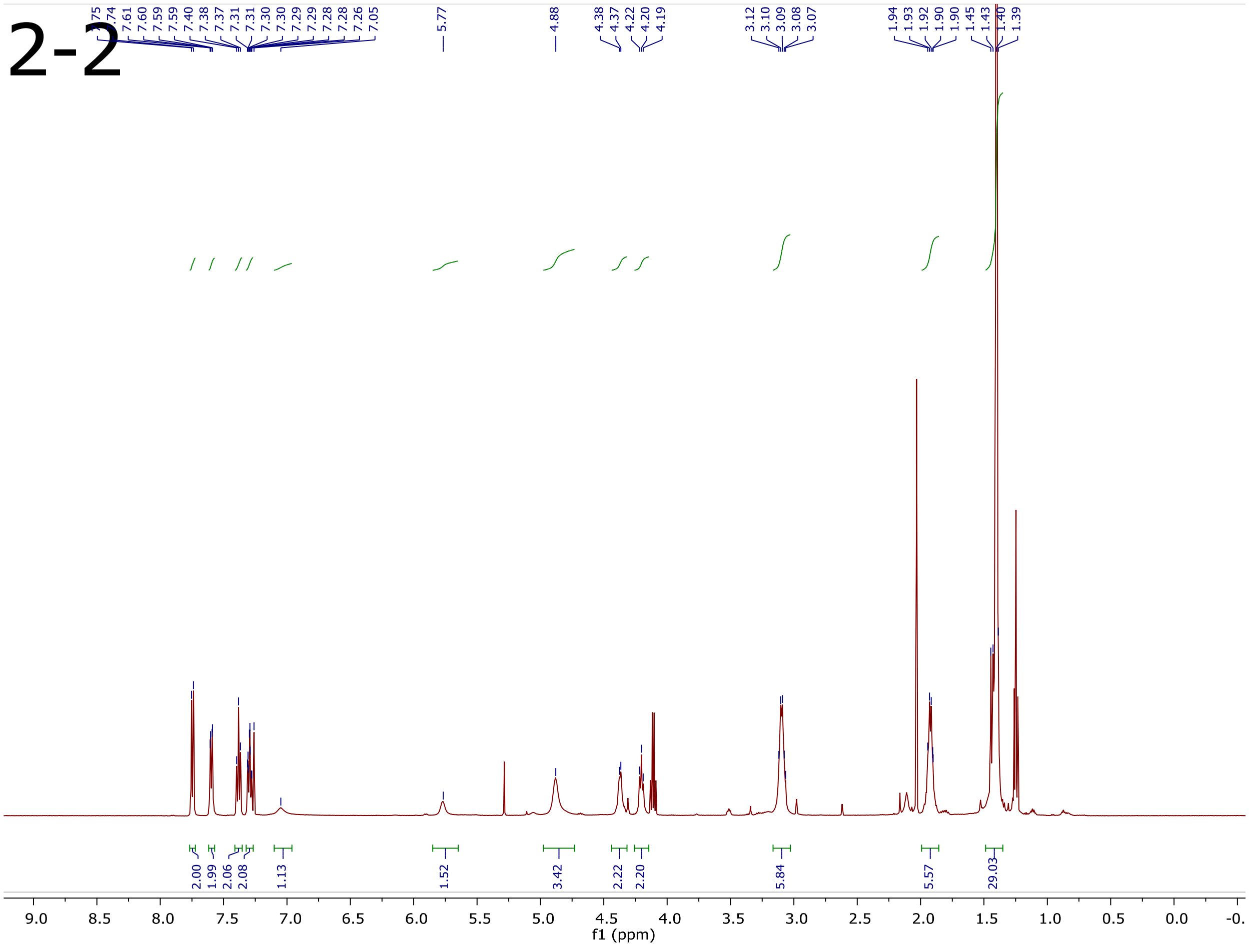

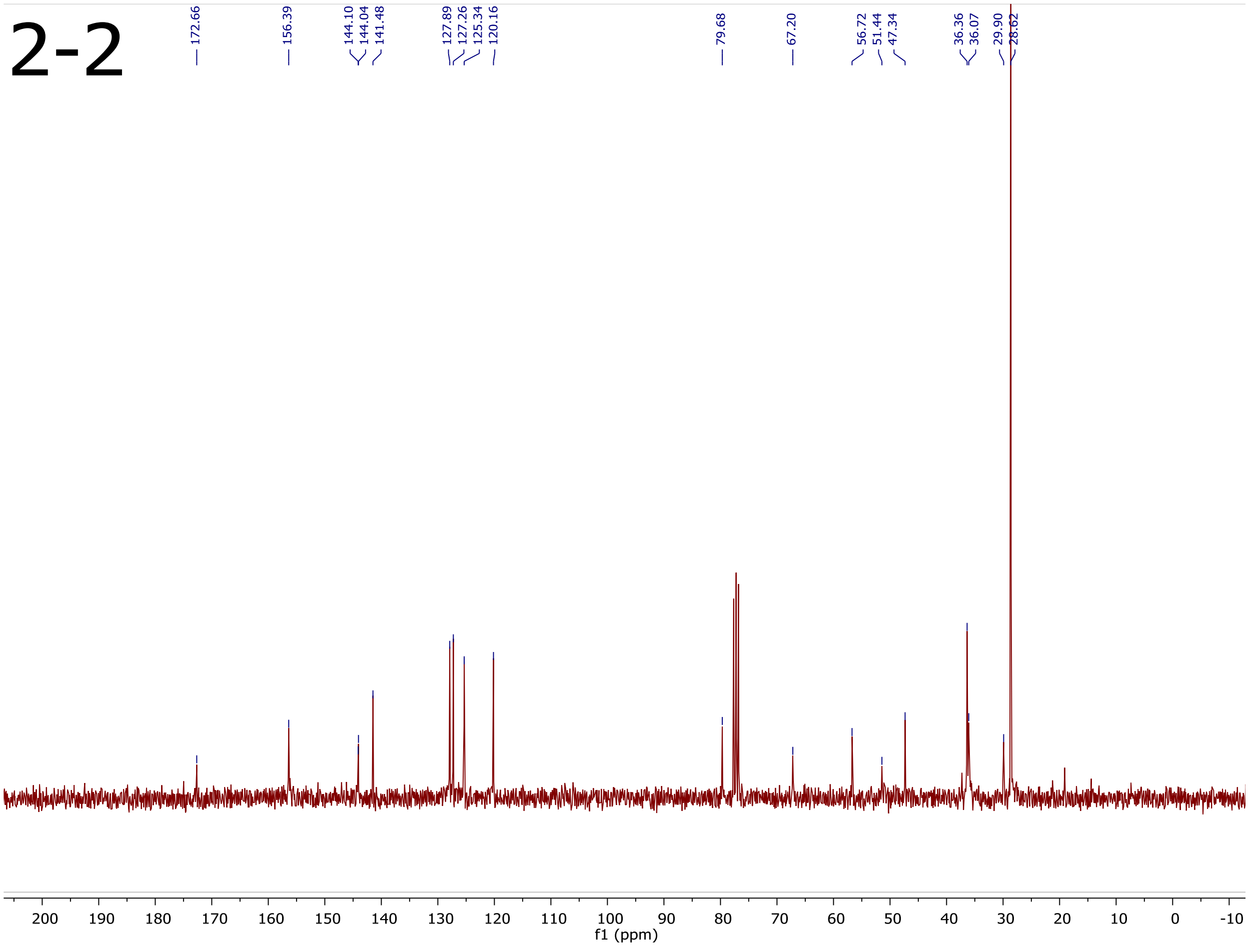
2-2
172.66
156.39
144.10
144.04
141.48
127.89
127.26
125.34
120.16
79.68
67.20
56.72
51.44
47.34
36.36
36.07
29.90
28.62
200 190 180 170 160 150 140 130 120 110 100 90 80 70 60 50 40 30 20 10 0 -10
f1 (ppm)

2

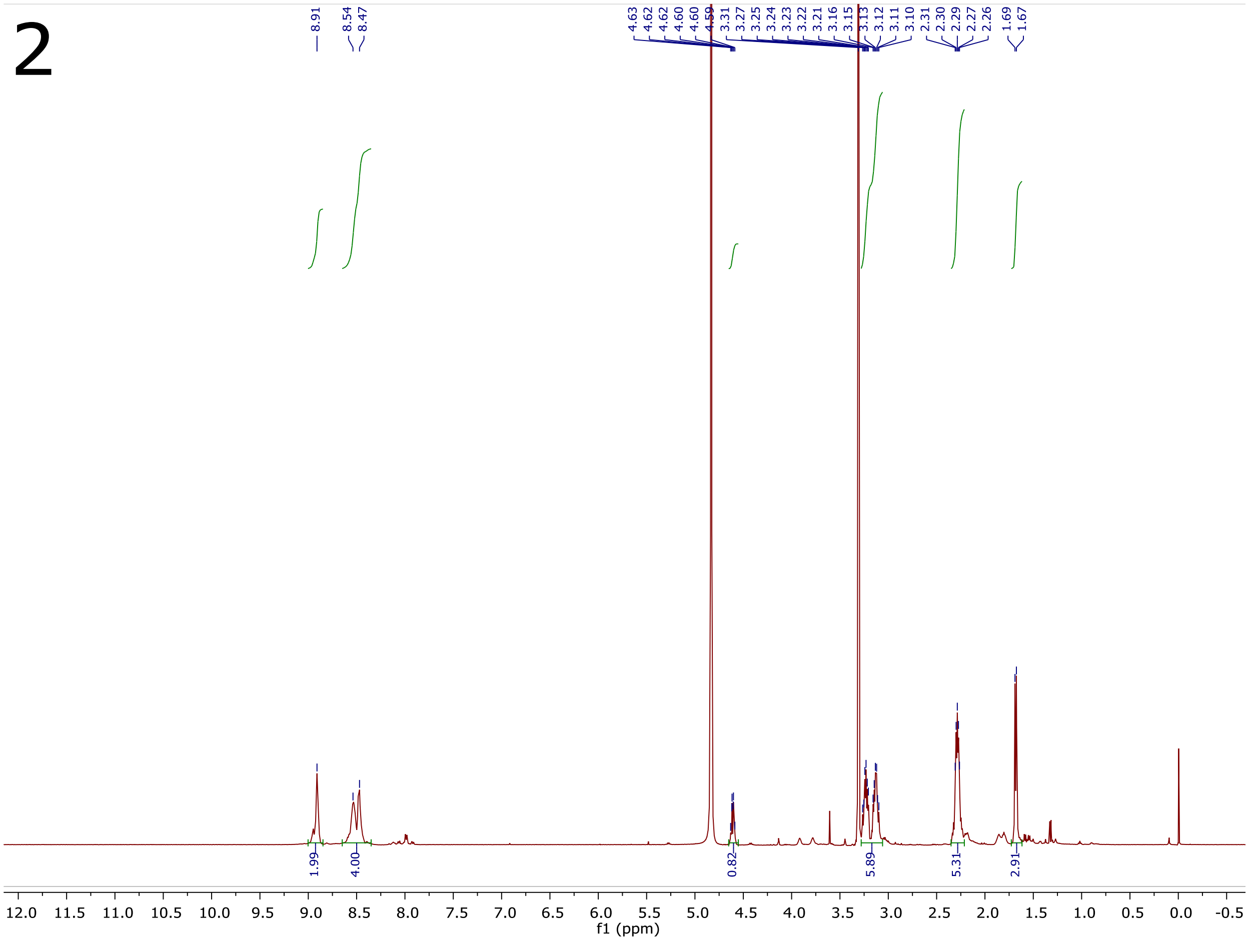
8.91
8.54
8.47
4.63
4.62
4.62
4.60
4.60
4.59
3.31
3.27
3.25
3.24
3.23
3.22
3.21
3.16
3.15
3.13
3.12
3.11
3.10
2.31
2.30
2.29
2.27
2.26
1.69
1.67
1.99
4.00
0.82
5.89
5.31
2.91
12.0 11.5 11.0 10.5 10.0 9.5 9.0 8.5 8.0 7.5 7.0 6.5 6.0 5.5 5.0 4.5 4.0 3.5 3.0 2.5 2.0 1.5 1.0 0.5 0.0 -0.5
f1 (ppm)

2

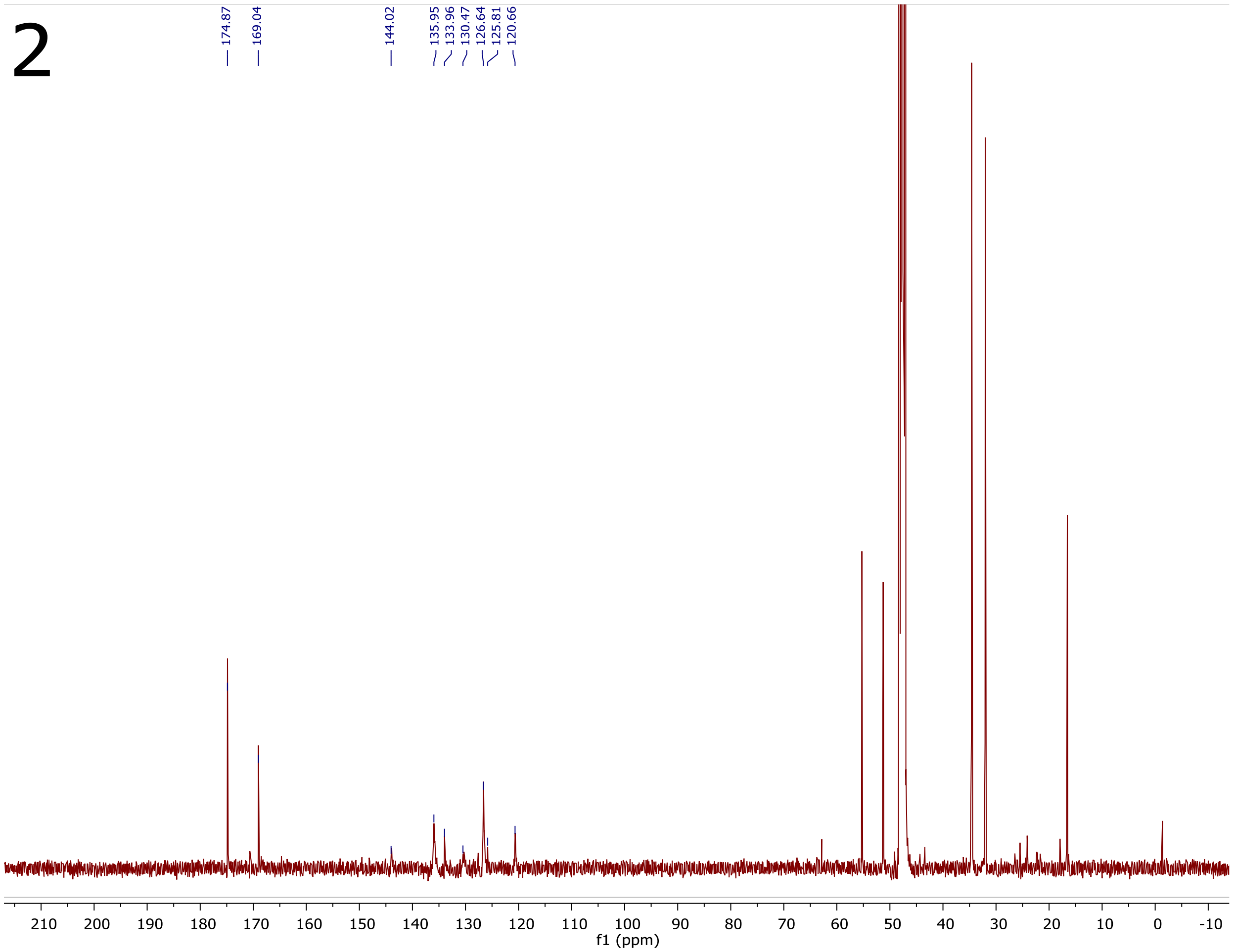

1. References

1. Amirbekyan, K.; Duchemin, N.; Benedetti, E.; Joseph, R.; Colon, A.; Markarian, S. A.; Bethge, L.; Vonhoff, S.; Klussmann, S.; Cossy, J. Design, Synthesis, and Binding Affinity Evaluation of Hoechst 33258 Derivatives for the Development of Sequence-Specific DNA-Based Asymmetric Catalysts. *ACS Catalysis* **2016,** *6*, 3096-3105, 10.1021/acscatal.6b00495.
2. Zhao, X.; Schanze, K. S. Fluorescent Ratiometric Sensing of Pyrophosphate Via Induced Aggregation of a Conjugated Polyelectrolyte. *Chem. Commun.* **2010,** *46*, 6075-6077, 10.1039/C0CC01332C.
